# New Work by Dr. F. Campbell Stewart

**Published:** 1843-07-08

**Authors:** 


We learn, with much pleasure, that Dr. F. Campbell Stewart, of New York, is about publishing "A General, Historical, and Statistical Account of the Hospitals of Paris, with Biographical Notices of the Most Eminent of the Parisian Surgeons." The work will be divided into two parts. The first part will contain the lives of the most distinguished surgeons,—as Velpeau, Roux, Jules Cloquet, Lisfranc, Civiale, &c.&c. The second part will embrace a historical and statistical account of the Public Hospitals, with useful and interesting information with regard to the School of Medicine, the expense of education and living in Paris, the Royal Academies, Medical Journals, &c.

A work of this kind has long been wanted. The

American Student goes abroad knowing little of the characters of the men he is to come in daily contact with; and totally ignorant what he is to do when he gets there. Much time is lost, and money expended in gaining experience. The present book will abridge materially this labour, and spare him much trouble and unnecessary expenditure. The biographical portion will be highly interesting to the profession in general.

Dr. Stewart was for some years a resident practitioner of medicine in Paris, and was attached to our embassy in a medical capacity. The extensive opportunities he enjoyed, and his high abilities (which are well known to our readers, from his contributions to this Journal,) are sufficient guarantee that the work will be ably executed. It is to be published by subscription, ($2.00.) Carey & Hart, and Grigg & Elliott, are the agents in this city.

